# Speech error rates after a sports-related concussion

**DOI:** 10.3389/fpsyg.2023.1135441

**Published:** 2023-03-07

**Authors:** Sona Patel, Caryn Grabowski, Vikram Dayalu, Anthony J. Testa

**Affiliations:** ^1^Department of Speech-Language Pathology, Seton Hall University, Nutley, NJ, United States; ^2^Department of Medical Sciences, Hackensack Meridian School of Medicine, Nutley, NJ, United States; ^3^Center for Sports Medicine, Seton Hall University, South Orange, NJ, United States

**Keywords:** sports-related concussion, concussion, mild traumatic brain injury, speech, fluency, disfluencies, speech error rate, pausing

## Abstract

**Background:**

Alterations in speech have long been identified as indicators of various neurologic conditions including traumatic brain injury, neurodegenerative diseases, and stroke. The extent to which speech errors occur in milder brain injuries, such as sports-related concussions, is unknown. The present study examined speech error rates in student athletes after a sports-related concussion compared to pre-injury speech performance in order to determine the presence and relevant characteristics of changes in speech production in this less easily detected neurologic condition.

**Methods:**

A within-subjects pre/post-injury design was used. A total of 359 Division I student athletes participated in pre-season baseline speech testing. Of these, 27 athletes (18–22 years) who sustained a concussion also participated in speech testing in the days immediately following diagnosis of concussion. Picture description tasks were utilized to prompt connected speech samples. These samples were recorded and then transcribed for identification of errors and disfluencies. These were coded by two trained raters using a 6-category system that included 14 types of error metrics.

**Results:**

Repeated measures analysis of variance was used to compare the difference in error rates at baseline and post-concussion. Results revealed significant increases in the speech error categories of pauses and time fillers (interjections/fillers). Additionally, regression analysis showed that a different pattern of errors and disfluencies occur after a sports-related concussion (primarily time fillers) compared to pre-injury (primarily pauses).

**Conclusion:**

Results demonstrate that speech error rates increase following even mild head injuries, in particular, sports-related concussion. Furthermore, the speech error patterns driving this increase in speech errors, rate of pauses and interjections, are distinct features of this neurological injury, which is in contrast with more severe injuries that are marked by articulation errors and an overall reduction in verbal output. Future studies should consider speech as a diagnostic tool for concussion.

## 1. Introduction

Sports-related concussion (SRC) occurs as a result of impact to the head or neck during competitive or recreational athletic activities (Powell et al., 2021). SRC is a specific classification of concussion or mild traumatic brain injury (mTBI), broad terms used to define milder forms of traumatic brain injury (TBI) which result from insult in a closed-head nature due to linear and/or rotational forces to the head or neck (McCrory et al., 2013). SRCs are reported to occur at a rate of 4.13 per 10,000 athlete exposure (Chandran et al., 2022). SRCs are typically characterized by a range of physical and cognitive symptoms, often initially determined by self-report of symptoms (McCrory et al., 2017). There are clinical factors that differentiate SRC as a less substantial injury than mTBI, such as an abbreviated recovery period, typically less than 10 days, as compared to mTBI, where recovery can take 2–4 weeks (King, 2019). In contrast with more severe brain injuries which cause visible abnormalities detectable through neuroimaging, the nature and relative severity of damage in SRCs make detection difficult through currently available methods of neuroimaging. Traumatic brain injury is typically classified in terms of severity using the Glasgow Coma Scale and other standardized scales. A concussion can be similarly graded according to scales such as the Nelson grading system (Nelson et al., 1984) or the Colorado Grading System (Ommaya, 1985), however, 90% of concussions do not involve loss of consciousness and under 24 h of amnesia, resulting in a grading of 2 or less on the Nelson and 1 on the Colorado (Cantu, 2006). Detection and diagnosis therefore rely on a series of assessments spanning a host of domains including measures of physical symptoms (e.g., balance, visual disturbances, headache, fatigue) as well as some basic cognitive functions (e.g., concentration, memory; Broglio et al., 2014; Echemendia et al., 2017). Variance across domains can occur and must be appraised in aggregate and interpreted by the clinical provider to determine the presence of injury, often in relation to broad normative data. To date, no practically available diagnostic marker for concussion exists.

One possible diagnostic marker for concussion is speech errors, characterized by deviations in timing, articulatory precision, and fluency (Darley et al., 1969). The neurologic underpinnings of speech production cover an expansive range of structures and related functions within the brain, involving “feed-forward” and “feedback” pathways that drive the conversion of cognitive-linguistic thought to motor planning and ultimately to speech-motor movements (Murdoch, 2001; Wildgruber et al., 2001; Guenther, 2006; Hickok, 2012). This complex neural circuitry constantly monitors and updates speech output through internal and external feedback loops, optimizing accuracy of production with minimal speech errors in neurologically healthy speakers (Fox Tree, 1995). On the other hand, brain injury and advanced diseases can impact cognitive and sensorimotor components of the speech production process and result in a substantial increase in the number of errors when speaking.

Speech errors are widely accepted as hallmark sequelae of neurotrauma in conditions such as stroke, brain injury, amyotrophic lateral sclerosis (ALS), Parkinson’s disease (PD), and multiple sclerosis (Yorkston, 1996; Hartelius et al., 2000; Tomik and Guiloff, 2010; Moro-Velazquez et al., 2021). These conditions can result in alterations in acoustic properties (Holmes et al., 2000; Rusz et al., 2011), articulatory precision (Karlsson and Hartelius, 2019; Karlsson et al., 2020), and measures of timing (Juste et al., 2018). The patterns of speech errors that occur can often predict the specific neurological condition, holding potential for use as biomarkers of various conditions of the central and peripheral nervous system. For example, various parameters of speech production have been identified as markers for individuals with focal cerebrovascular accidents, commonly referred to as “stroke.” Individuals with right hemisphere stroke typically exhibit significant reductions in fundamental frequency range, which is especially apparent when expressing emotional tones, mainly “joy” and “anger” (Ross and Monnot, 2008; Guranski and Podemski, 2015; Patel et al., 2018). In addition, the prosodic quality of stress, comprised of multiple acoustic factors including pitch, intensity, vowel quality, and duration (Chrabaszcz et al., 2014), have been found to indicate cortical hemispheric effects. Balan and Gandour (1999) identified limitations in the ability of individuals with right hemisphere stroke to shift or adjust stress to the same degree as health controls. In addition, Vergis et al. (2014) identified significant difference in speech rate and vowel duration among individuals with left hemisphere stroke resulting in apraxia of speech and aphasia when compared to those with aphasia alone as well as healthy controls.

The specific neurological impacts of TBI vary based on the location and nature of the injury, including possible etiologies of hematoma, hemorrhage, and diffuse axonal injury (Mesfin and Taylor, 2017). Severe and moderate TBIs result in symptoms of motor speech impairment, such as dysarthria (Goozée et al., 2001; Solomon et al., 2001; Wang et al., 2005; McAuliffe et al., 2010; Kuruvilla et al., 2012) and occasionally apraxia of speech (Yadegari et al., 2014). Common characteristics include a slower articulation rate, smaller proportion of phonation time relative to sample duration, and larger total pause time (Wang et al., 2005). Other research in severe TBIs using analysis of passage readings has identified deficits in rate, resonance, and precision of consonants/overall intelligibility, variations in pitch and general stress patterns, as well as changes in phrase length among other aspects of speech production compared to healthy controls (Theodoros et al., 1994). Recent research with severely injured young children (6–10 years) suggests that during conversations there are decreases in pitch variation, the number of unique phonemes spoken, pause lengths, and increased variability of articulation rate (Noufi et al., 2019). All such findings indicate that speech deficits are a strong indicator of the presence and severity of TBI.

Deviations in various elements of speech production have also been identified as possible markers of injury in advanced stages of Alzheimer’s disease, a disease process resulting from deviations in neural cellular health and integrity associated with abnormal protein deposits and metabolic processing ultimately resulting in diffuse failure of brain health and function (Mohandas et al., 2009). Speech characteristics of individuals with Alzheimer’s disease include temporal changes, such as reduced rate of speech (phoneme or syllable production), increased pause or hesitation ratio, increased instances of repetitions, and increased frequency of within and between phrase pauses (Hoffmann et al., 2010; Fraser et al., 2016; Pistono et al., 2016; Slegers et al., 2018). Even in milder or earlier stages of neurodegeneration, such as early-stage Alzheimer’s disease or mild cognitive impairment, differences in speech characteristics exist when compared to neurologically healthy controls. Analysis of connected speech samples in individuals with mild cognitive impairment has revealed alterations in articulation rate with and without hesitations, silent pauses, hesitation ratio, length of utterances, and pause per utterance when compared to healthy controls (Tóth et al., 2018).

Despite the scaled parallel in physical and cognitive symptoms commonly identified in concussion and more severe head injuries, consideration of the impacts of concussion on speech production has been limited. Changes in speech are typically not captured on commonly used symptom inventories for milder injuries and sports-related sideline assessments (Schatz et al., 2006; Asken et al., 2020). However, recent early evidence has shown significant alterations in rate of speech (Salvatore et al., 2019), acoustic features (Daudet et al., 2017), articulatory precision (Chong et al., 2021) and fluency (Robertson and Diaz, 2020; Rose et al., 2021; Toldi and Jones, 2021) in concussion. These preliminary findings suggest that further examination of speech changes in milder head injuries is necessary in a larger sample in order to establish the specific pattern of speech changes associated with concussion.

The goal of this study was to identify the speech changes that occur following an SRC using a comprehensive system for coding errors. Because error rates (disfluencies, misarticulations, speech errors) in typical speech production are low, small deviations from normal that might occur after a concussion may not be noticeable or identified as disordered because the errors do not interfere with functional communication, even though these errors may be systematically or consistently occurring. To investigate whether small deviations in speech fluency or patterns of speech errors exist, the present study analyzed speech samples of student athletes obtained in the days immediately following a concussion and compared these samples to their individual baseline recordings obtained prior to injury. A picture description task was used to obtain a more ecologically valid assessment of speech errors and disfluencies present. We expected student athletes with SRC to demonstrate an overall increase in the total number of speech errors compared to their individual pre-injury levels. Further, we anticipated observable patterns of errors that resemble those of more severe head injuries, albeit reduced in frequency.

## 2. Materials and methods

### 2.1. Participants

From 2018 through 2021, consenting Division I student athletes at Seton Hall University (*n* = 359) underwent speech testing concurrent with baseline testing that is completed annually as standard of care by Sports Medicine. All participants were proficient in English in lines with academic demands. All participants reported no history of vision, hearing, speech, or language issues, neurological disorders, or diagnosed psychiatric disorders (e.g., anxiety, depression, bipolar disorder). Additionally, it was confirmed at intake that participants were not experiencing upper or lower respiratory infections or other conditions that would impact speech and voice quality at the time of testing. Of the individuals tested, 27 athletes (11 males, 16 females; mean age: 18.3 years, range of 18–25 years) were determined to have a concussion by a Certified Athletic Trainer from Seton Hall University’s Sports Medicine. All of the athletes diagnosed with SRC in this study had 0 min of loss of consciousness and under no reported amnesia. Injured participants represented eight sports teams at the University (see Table 1). All injured participants were initially evaluated as per the Sports Medicine protocol and were referred for testing once presence of concussion was determined. In some cases, due to latency of symptom onset or evolving presentation (such as headaches, light or sound sensitivity, sleep disturbance, among others), confirmation of the presence of concussion occurred up to 36 h after injury (Ruff et al., 2009). Participants with concussion then underwent post-injury speech testing, matching baseline testing procedures. All participants provided informed consent in accordance with the Hackensack Meridian Health Institutional Review Board on behalf of Seton Hall University.

**Table 1:** Demographic information of participants who sustained a concussion, including age, sex, sport, time of testing post-injury (days), and scores on the Standardized Assessment of Concussion (SAC).

Subject	Age	Sex	Sport	Days post-injury	SAC post-injury
s21	22	m	Men’s Basketball	2	25
s38	19	f	Women’s Soccer	1	28
s40	20	f	Women’s Soccer	6	27
s45	23	m	Men’s Soccer	2	26
s49	19	m	Men’s Soccer	2	28
s61	18	m	Men’s Soccer	<24 h	25
s63	18	f	Women’s Soccer	4	25
s64	21	f	Women’s Soccer	2	27
s73	21	m	Men’s Soccer	4	28
s78	22	m	Men’s Soccer	2	27
s108	19	f	Softball	3	29
s102	18	f	Women’s Basketball	4	19
s103	22	f	Women’s Basketball	1	26
s104	18	f	Women’s Golf	2	23
s116	22	f	Women’s Basketball	2	29
s141	20	f	Women’s Soccer	4	24
s144	20	m	Baseball	5	29
s219	18	m	Baseball	3	26
s227	20	f	Women’s Basketball	3	26
s231	18	f	Women’s Basketball	3	28
s233	24	m	Men’s Soccer	2	27
s241	20	f	Women’s Soccer	6	27
s244	25	m	Men’s Soccer	2	22
s248	18	m	Baseball	2	23
s270	19	f	Softball	1	29
s315	20	f	Volleyball	3	28
s321	18	f	Volleyball	3	29

### 2.2. Procedures

This study used a pre-test/post-test design where the same speech and language tasks were performed by participants before and after injury. Each test session was completed in a quiet study room reserved for student-athletes in under 20 min. As a part of baseline testing, all participants completed an intake questionnaire at the time of consent that included questions pertaining to demographic information and relevant medical history. Injured participants were tested in the days after being diagnosed with a concussion (mean = 2.83 days; range 0–6 days; see Table 1). Table 1 also provides the Standardized Assessment of Concussion (SAC) score post-injury as an indicator of concussion (out of 30; McCrea et al., 1998). Testing included a variety of speech elicitation tasks ranging in complexity and duration. Speech was recorded using an AKG head-worn microphone (HARMON International, Stamford, CT), which was routed through an Apollo audio interface with preamplifier (Universal Audio, Inc., Scotts Valley, CA) that was connected to a laptop computer dedicated for speech data collection. Audition software (Adobe, San José, CA) was used to record and store speech signals as.wav files onto the computer. Here we examined the audio files collected from one of the testing tasks, specifically the standard picture description task where participants were presented with a visual stimulus featuring a scene with multiple elements to elicit verbal output (e.g., “The Cookie Theft”; Goodglass, 1983; Shimada et al., 1998). Participants were instructed by the experimenter to “Take a look at this picture and explain to me what is happening. Tell me everything you can about the picture.”

### 2.3. Speech error coding

To prepare the sound files for analysis (27 pre-injury or baseline samples, 27 post-concussion samples), extraneous speech that indicated acknowledgement of the task (e.g., “Okay”) or the end of one’s description (e.g., “That’s about it”) was removed. All sound files were transcribed in order to compute the number of syllables per sample. Next, error analysis was performed by two coders, who listened to the sound files to identify speech disfluencies and errors. Speech disfluencies and errors were classified as 1 of 14 types based on a combination of coding procedures commonly utilized in fluency and speech analysis (Lutz and Mallard, 1986; St. Louis et al., 1991; Ambrose and Yairi, 1999; Shriberg, 2001; Roberts et al., 2009; Sawyer and Yairi, 2010; Duffy, 2019) and marked on the transcript. Both coders were trained to identify and code 14 error types based on the specific definitions noted in Table 2. These 14 error types were also collapsed into 6 major categories based on shared features: pauses, revision/incomplete utterances, repetitions, articulation errors, time fillers, and prolongations. For example, all errors featuring repeated speech output were grouped into one larger category of “repetition” errors; all sound-level errors were grouped into the larger category of “articulation errors.” Both coders converged on the location and type of each error. The coders were blinded to the subject and condition when coding the speech samples. Reliability was assessed on approximately 15% of subjects (Corey and Cuddapah, 2008). Inter-rater reliability was calculated as the Pearson’s correlation between raters for each error category. Inter-rater reliability across error categories was acceptable (greater than.81; McHugh, 2012): total errors = 0.98, articulation = n/a (no errors across samples tested), prolongation = 0.86, pause = 0.94, time fillers = 0.98, revision/incomplete = 0.92, and repetition = 1.0.

**Table 2:** Coding criteria for speech errors and disfluencies within six major error categories.

Error/Disfluency category	Definition
Articulation	*Sound-level errors in articulation that include distortions, additions, omissions, substitutions*
Substitution	Any sound substitution
Distortion	Any sound-level distortion
Addition	A sound that is added
Omission	A sound that is omitted from a word
Pause	*Pauses greater than 250 ms*
Prolongation	*Sounds or syllables extended in duration more than 250 ms*
Repetition	*Any utterance (sound, word, phrase) that is repeated*
Part-word	Repetition of one or more phonemes within a word
Single-syllable whole-word	Repetition of a single syllable word
Multisyllable whole-word	Repetition of a word with two or more syllables
Phrase	Repetition of a phrase, i.e., a connected string of words
Revision/Incomplete	*A change or correction of an utterance(s) that did not convey a complete thought*
Revision	Modifications to output at a syllable, word, or phrase level
Incomplete segment	Utterance terminated abruptly or does not convey a complete thought
Time Fillers	*Extraneous sounds, words, or phrases that do not contribute to the meaning of the utterance*
Interjection	Words/phrases that are syntactically appropriate but do not add to the intended message (e.g., “So you know…,” “I guess”)
Filler	Sounds or “non-words” that add no meaning to the intended message (e.g., “um” and “uhh”)

### 2.4. Statistical analyses

Error rates were computed for each speech error type of each sample in order to normalize error totals to the amount of speech produced (number of errors divided by the number of syllables). Since this study sought to examine changes in the within-subjects factor of time (baseline, concussion), a repeated measures analysis was required. Kolmogorov-Smirnoff tests of normality were significant (*p* < 0.05) for all parameters except fillers and pauses and the larger categories of time fillers, total dysfluency, and number of syllables at baseline. Results were similar after concussion, in addition to a lack of significance (*p* > 0.05) for interjections, indicating deviations from normality for most parameters. Examination of the skewness and kurtosis values revealed larger values than the standard error for either the baseline or concussion data for each parameter, indicating that assumptions of homoscedasticity also appear to have not been fully met. Thus, non-parametric Friedman tests were performed in SPSS (IBM SPSS Statistics v.28, Chicago, IL) on the error rates for the number of syllables produced, the total error rate, each of the six major error categories, and the 14 individual error types.

Next, stepwise regressions with bidirectional selection were performed on the 14 error types separately at baseline and after concussion to determine the extent that these variables best captured the overall error rate. Bidirectional selection involves a mixture of the forward and backward procedures in which the variable that explained the most variance in the total error rate was entered into the model first (entry criteria: probably of *f* = 0.05), followed by the variable that explained most of the residual variance, resulting in a set of variables with the largest regression coefficients for inclusion in the model (Snyder, 1991). Such procedures can be advantageous for identifying the primary contributors when the number of parameters is small, thus resulting in a model with the smallest number of variables (Lewis, 2007). At each step, the predictors that were no longer significant were removed (removal criteria: probability of *f* = 0.1). Variables that accounted for more than 3% of the variance in the total error rate are reported.

## 3. Results

**Figure 1:**
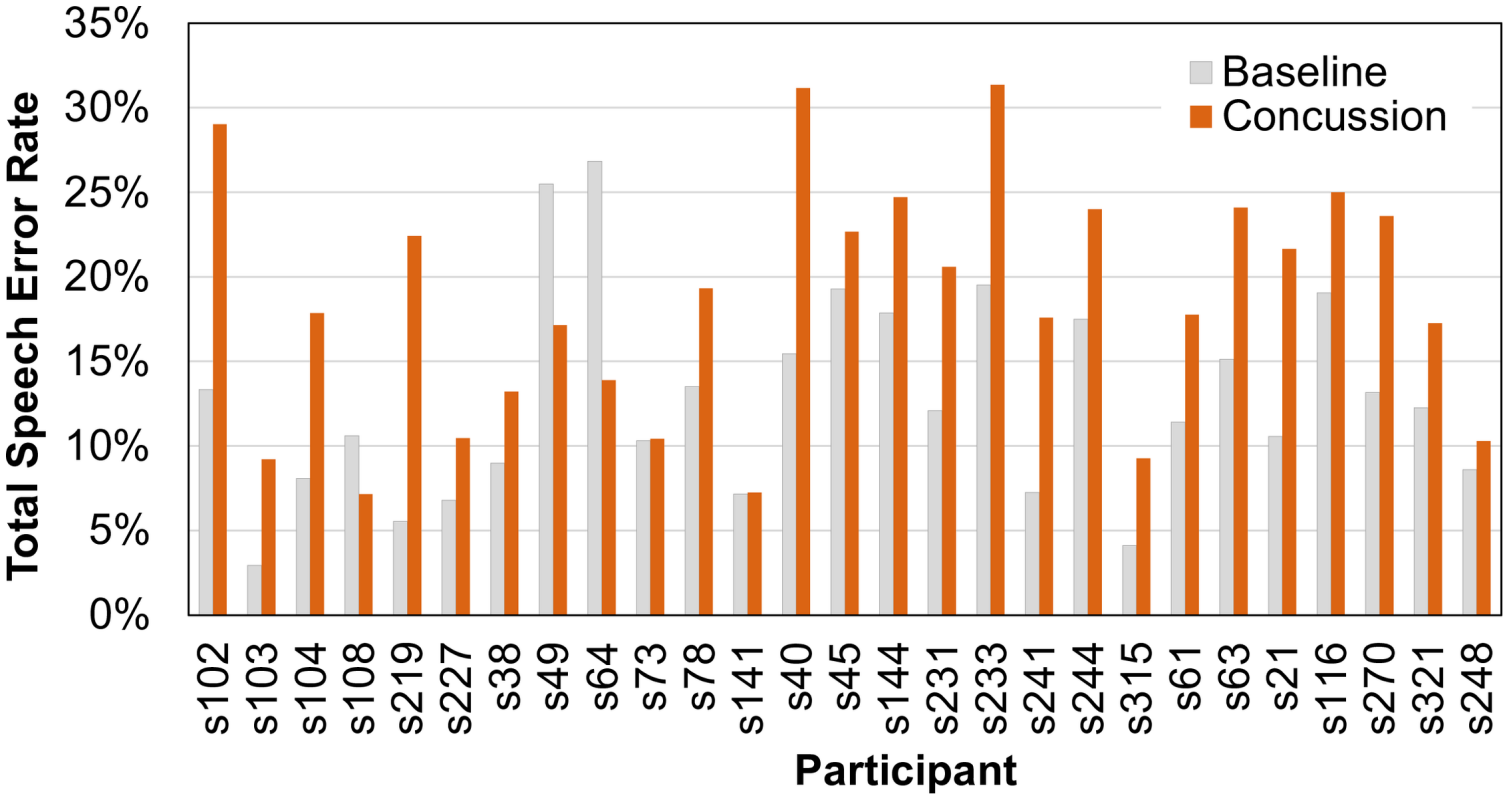
Total speech error rates (percent) for individual participants before and after a sports-related concussion.

Results showed no significant difference in the average number of syllables produced after a concussion (mean or *M* = 99.4; standard deviation or SD = 43.4) compared to baseline (*M* = 97.7; SD = 41.1) at the *α* = 0.05 level (*Χ^2^* = 0.333, *p* = 0.564). Nevertheless, individual differences in the number of syllables produced by each person existed. Therefore, the number of errors within each error type were normalized to the number of syllables, resulting in an error rate for each error type. The total error rate was significantly different between baseline and concussion samples (*Χ^2^* = 16.333, *p* < 0.001). The percentage of speech errors increased after sustaining a concussion (*M* = 18.5%; SD = 0.07) compared to baseline (*M* = 12.7%; SD = 0.06). Individual pre- and post-injury error scores are shown for each participant in Figure 1.

**Figure 2:**
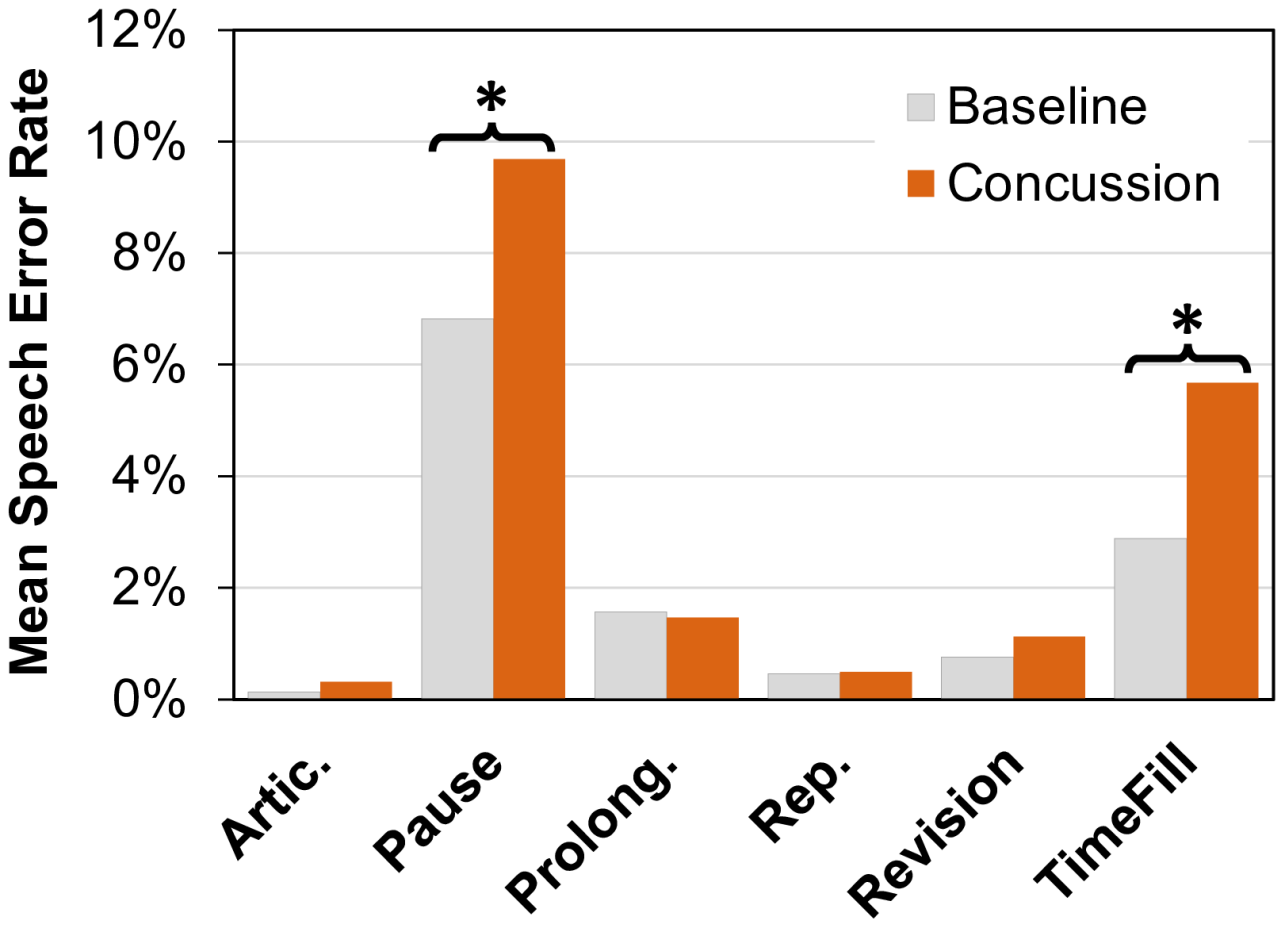
Mean speech error rate (percent) for articulation errors (Artic.), pauses, prolongations, (Prolong.), repetitions (Rep.), revisions, and time fillers (TimeFill) at baseline and after concussion. A significant difference between conditions (*α* = 0.05 level) is indicated by an asterisk (*).

Next, changes in the number of errors and disfluencies in the six error categories were examined (see Figure 2). Friedman tests of the error rates showed significant differences (*α* = 0.05) in the pause category (*Χ^2^* = 10.704, *p* = 0.001) and the time filler category (*Χ^2^* = 19.593, *p* < 0.001), with higher error rates occurring after a concussion (pauses: *M*_pre_ = 6.8%, *M*_post_ = 9.7%; time fillers: *M*_pre_ = 2.9%, *M*_post_ = 5.7%). No significant changes were found in articulation errors (*Χ^2^* = 2.778, *p* = 0.096), prolongations (*Χ^2^* = 1.190, *p* = 0.275), repetitions (*Χ^2^* = 0.111, *p* = 0.739), or revisions/incompletes (*Χ^2^* = 0.25, *p* = 0.617).

As the time filler, articulation error, repetition, and revision/incomplete categories consisted of multiple parameters, additional Friedman tests were performed to examine changes in particular error types. Results showed significant differences in the rate of interjections after a concussion (*p* < 0.05). The change in fillers approached significance (*p* = 0.072). Results are shown in Table 3.

**Table 3:** Results of Friedman tests of speech error/disfluency rates at the *α* = 0.05 level for baseline compared to concussion.

Error/Disfluency category	df	Χ*^2^*	*p*
Articulation	1	2.778	0.096
Substitution	1	–	–
Distortion	1	2.667	0.102
Addition	1	0	1.000
Omission	1	2.000	0.157
Pause	1	10.704	0.001*
Prolongation	1	1.190	0.275
Repetition	1	0.111	0.739
Part-word	1	1.286	0.257
Single-syllable whole-word	1	2.667	0.102
Multisyllable whole-word	1	1.000	0.317
Phrase	1	0.333	0.564
Revision/Incomplete	1	0.25	0.617
Revision	1	1.471	0.225
Incomplete segment	1	0.333	0.564
Time Fillers	1	19.593	<0.001*
Interjection	1	22.154	<0.001*
Filler	1	3.240	0.072

^*^There were no occurrences of sound substitutions for any subject.

Stepwise regressions of the 14 error types at baseline showed that the total error rate was primarily driven by pauses [*R*^2^ = 0.628, ΔF(1,25) = 42.162, *p* < 0.001]. Prolongations accounted for an additional 28.1% of the variance [*R*^2^ = 0.909, ΔF(1,24) = 74.260, *p* < 0.001] followed by time fillers, which accounted for an additional 3.1% of the variance [*R*^2^ = 0.940, ΔF(1,23) = 12.130, *p* = 0.002]. In contrast, the total error rate after a concussion was primarily driven by time fillers [*R*^2^ = 0.707, ΔF(1,25) = 60.252, *p* < 0.001]. Pauses accounted for an additional 18.4% of the variance [*R*^2^ = 0.891, ΔF(1,24) = 40.419, *p* < 0.001] followed by prolongations, which accounted for an additional 5.3% of the variance [*R*^2^ = 0.944, ΔF(1,23) = 21.882, *p* < 0.001]. Addition of a fourth variable accounted for less than 3% of additional variance. Despite significant *p*-values for additional parameters, overfitting of models can produce misleading results. We decided to exclude variables that were contributing 3% of the variance to avoid over-fitting and simply report on the major factors contributing to the model. Such procedures have been used in prior work (Patel and Shrivastav, 2011).

## 4. Discussion

Changes in the characteristics of speech production including the presence of errors or deviations from typical are known to occur across various neurological conditions, including moderate and severe brain injury. However, it is not known whether the patterns of speech changes that occur in milder forms of brain injury such as SRC resemble more severe forms of brain injury or whether distinct errors patterns reflective of SRC exist. In this study we examined speech error patterns in Division I college athletes within 6 days following a SRC, with a prediction that the total number of speech errors and dysfluencies would increase after a concussion compared to individual pre-injury levels. The availability of individual baseline measures is uncommon in brain injury and is advantageous as it allows more sensitive identification of error trends. As predicted, within-subject comparisons demonstrated a significant increase in the speech error rate after an SRC. To the best of our knowledge, this is the first study to demonstrate increases in speech error rates using a comprehensive system for capturing errors across domains of articulation, fluency, and timing in a large sample.

Our second prediction was that error types in SRC would be similar to those in more severe TBIs, specifically in the manifestation of articulation errors, reduced verbal output, and increased frequency of pausing (Power et al., 2020). In order to evaluate this prediction, errors were coded based on a classification system comprised of six major categories representing a total of 14 error types. Two of the six error categories, namely, number of pauses and time fillers, increased significantly after a concussion. The “pause” category captured any period of silence greater than 250 ms. Previous research has shown an increase in pausing, particularly pause duration, in TBI and even in health individuals under conditions of increased cognitive demand (Wang et al., 2005; Khawaja et al., 2008; Noufi et al., 2019). In line with these findings, the results of the present study in SRC showed that the number of pauses increased in milder head injuries. One other study by Banks et al. (2021) showed a similar pattern of results using a different task, namely that the time interval between syllables in a diadochokinetic speech task (repeated syllable production) was longer than the time interval in healthy controls. Despite the observed increase in the number of pauses in the present study, the number of syllables produced after a concussion was not significantly different from pre-injury baselines. In other words, the increased number of pauses contributed to a lengthening of the overall duration of the speech sample without a reduction in the total verbal output. These findings are in contrast with TBI research (more severe injuries than SRC) that shows a decrease in utterance length (Stubbs et al., 2018).

Although the total number of syllables did not reduce after an SRC, the verbal output might have been reduced in overall complexity, as indicated by a significant increase in the number of “time fillers” in the present study. The “time fillers” category examined in the present study included two error types that capture additional sounds, words or phrases that do not contribute to the sentence structure or meaning, namely interjections and fillers (see Table 1). Fillers differ from interjections in that they are extraneous sounds or non-words (Corley and Stewart, 2008), while interjections are extraneous words or phrases. In the present study, the number of interjections significantly increased but not the number of fillers, although they were trending. In other words, fillers occurred frequently prior to injury and continued to increase after injury. Both error types functionally serve to maintain continuity of connected speech production while accommodating increased demands on planning intended speech (Clark and Fox Tree, 2002).

The distribution of errors was also examined in athletes before any head injury to determine whether the pattern of errors changed after SRC. Results from the regression analysis of the total speech error rate at baseline by the six error categories showed that pre-injury errors primarily consist of pauses, followed by prolongations. Pausing is a behavior that allows time for linguistic ideation, motor planning, and execution for coherent and fluent speech production and a certain number of pauses are expected to occur when speaking. Prolongations slow down the rate of speaking allowing for thinking while still creating continuity/connectivity in verbal output as time fillers do, but in a subtle, less disruptive manner, that can in many cases can be perceived as typical alterations in stress patterning that occur in discourse. In contrast, errors after an SRC were primarily time fillers, followed by pauses. This suggests that individuals with SRC use time fillers (particularly interjections) to allow for seamless transition of thoughts while speaking more frequently than silent periods (pausing). This finding showcases that individuals with SRC may manifest a unique set of compensatory mechanisms to deal with the underlying neural insult. Further, in comparison to speech error data from more severe brain injuries, the use of time fillers is a unique communicative e pattern that may be available only to individuals with milder concussions.

The increased number of pauses and time fillers in individuals with SRC suggests inefficiencies in the planning of linguistic content, which are rooted in the cognitive domains of attention, memory and higher order executive functions (King et al., 2006; Crawford et al., 2007; Murray, 2012; Obermeyer et al., 2020). Concussion is known to impact the areas of cognition associated with the planning of speech output, specifically executive functions, attention, and memory (Covassin and Elbin, 2010; Kaltiainen et al., 2019). Incidentally, these cognitive linguistic functions primarily occur in cortical regions where axonal sheering and other trauma occur in SRC (Shaw, 2002). It is therefore likely that the increased rate of pauses and time fillers identified in this study is an indication of underlying cognitive dysfunction.

The relationship of these speech error categories and cognitive linguistic function is seen in typical, non-injured adults, where the number of speech errors increases with higher processing, cognitive load, and cognitive ability (Bortfeld et al., 2001; Shriberg, 2001; Engelhardt et al., 2013). The relationship of cognitive impairment and speech errors has also been established across various clinical populations. Both Power et al. (2020) and Smith et al. (2018) have demonstrated that the time fillers category (interjections and fillers) is associated with cognitive deficits in adults with Parkinson’s disease. Other works have demonstrated higher-level relationships between speech output and cognitive-linguistic function, where individuals with left hemisphere stroke experience impairments in accessing the lexical-semantic network resulting in long pauses and decreased speech fluency (Yee et al., 2008; Lerman et al., 2020). In Alzheimer’s disease, studies note decreases in quantity of verbal output, decreases in richness of content, and increases in semantic errors as hallmark changes representative of the disease (Kavé and Levy, 2003; de Lira et al., 2014; Slegers et al., 2018). Milder forms of neurological decline such as mild cognitive impairment and early dementia have also shown impacts on verbal fluency and speech output, demonstrating differences in linguistic properties compared to healthy controls (Beltrami et al., 2018). The sum of findings across healthy individuals and those with various neurological conditions demonstrates that speech changes are linked to cognitive processes.

In the present study, the error categories of revision/incomplete, repetition, and prolongation did not show significant changes in SRC. The categories of revision/incomplete, repetition, and prolongation have been reported error types in TBI, although the incidence is extremely low and often connected with acquired stuttering disorders (Jokel et al., 2007). Some studies report stuttering-like behaviors, including speech hesitations, brief blocks, rapid repetitions, and occasional prolongations after TBI, in addition to interjections, silent pauses, broken words, revisions and starters (Lundie et al., 2014; Roth et al., 2015). These error categories, more stuttering-like in nature, may therefore be associated with more severe injuries or concomitant conditions (e.g., post-traumatic stress disorder) not typically present in SRC (Lundgren et al., 2010; Norman et al., 2018).

Finally, the error category of articulation also failed to show significant differences between baseline and concussion conditions. Articulation errors were predicted to contribute significantly to the error patterns in SRC as it is a common issue in more severe forms of neurotrauma and neurologic disease. Articulation errors are highly prevalent in TBI, as dysarthria, or speech dysfunction due to changes in muscle strength, range-of-motion, and coordination, occurs in up to 60% of individuals with TBI in acute phases of recovery (Yorkston, 1996). Most forms of dysarthria associated with articulatory imprecision and related errors result from insult to subcortical brain regions or peripheral nerve damage (Duffy, 2019). In the case of SRC it is therefore likely that mild cortical level trauma associated with axonal sheering does not yield impacts to the neuromuscular substrates of speech production that would amount to causing errors of articulation. This further supports the notion that speech errors found in SRC, pauses and time fillers, are associated with cognitive-linguistic dysfunction rooted in insult to cortical regions of the brain.

One limitation of the current study is the inclusion of the mildest of mild cases. In order to provide the best care for student athletes, the health team identified all possible cases of concussion that might have occurred. In other words, anyone who had sustained an impact to the head underwent sideline testing for symptoms of concussion. Speech evaluations were performed on all such cases, which may have resulted in a few referrals where symptoms of concussion were minimal. In the future, these will be controlled by setting a minimum symptom score as part of the inclusion criteria. In addition, future work should examine other factors that may influence changes in disfluency, including cognitive-linguistic function, severity of injury, etc.

## 5. Conclusion

The purpose of this study was to determine whether changes in speech error patterns exist in the days following a sports-related concussion compared to pre-season baseline measures. Our findings suggest the presence of increased speech errors in SRC. Specifically, significantly increased rates of pauses and time fillers were observed. Therefore, speech errors serve as a measurable marker of SRC that is not typically considered in current methods of clinical evaluation. The error patterns in the present study differ from the patterns of speech changes reported in the literature for other types of neurologic disorders including severe TBI. Thus, speech changes may serve to indicate the presence of concussion or milder forms of TBI. Further work must be done to understand the relationship of speech errors in SRC to higher-order cognitive functions as well as other symptom measures of SRC.

## Data availability statement

The raw data supporting the conclusions of this article will be made available by the authors, without undue reservation.

## Ethics statement

The studies involving human participants were reviewed and approved by Hackensack University Medical Center Institutional Review Board on behalf of Seton Hall University. The patients/participants provided their written informed consent to participate in this study.

## Author contributions

SP and AT contributed to the conception and design of this study. SP, AT, and CG contributed to the data collection. CG and SP wrote the first draft of the manuscript. VD and SP contributed to the design of the analysis. SP and CG performed the speech analysis. SP performed the statistical analysis. AT and VD provided feedback on different drafts of this manuscript. All authors contributed to the article and approved the submitted version.

## Funding

Research reported in this publication was partially supported by a New Jersey Commission on Brain Injury Research Pilot Grant (CBIR22PIL018) and an Undergraduate Research Award from Seton Hall University.

## Conflict of interest

The authors declare that the research was conducted in the absence of any commercial or financial relationships that could be construed as a potential conflict of interest.

## Publisher’s note

All claims expressed in this article are solely those of the authors and do not necessarily represent those of their affiliated organizations, or those of the publisher, the editors and the reviewers. Any product that may be evaluated in this article, or claim that may be made by its manufacturer, is not guaranteed or endorsed by the publisher.
